# Supplementary material for: Head and neck squamous cell carcinoma-specific prognostic signature and drug sensitive subtypes based on programmed cell death-related genes
Source: PeerJ. 2023 Nov 21;11:e16364. doi: 10.7717/peerj.16364 (PMC10668860; doi:10.7717/peerj.16364)
Supplement: Supplemental Information 1 [file peerj-11-16364-s001.pdf]

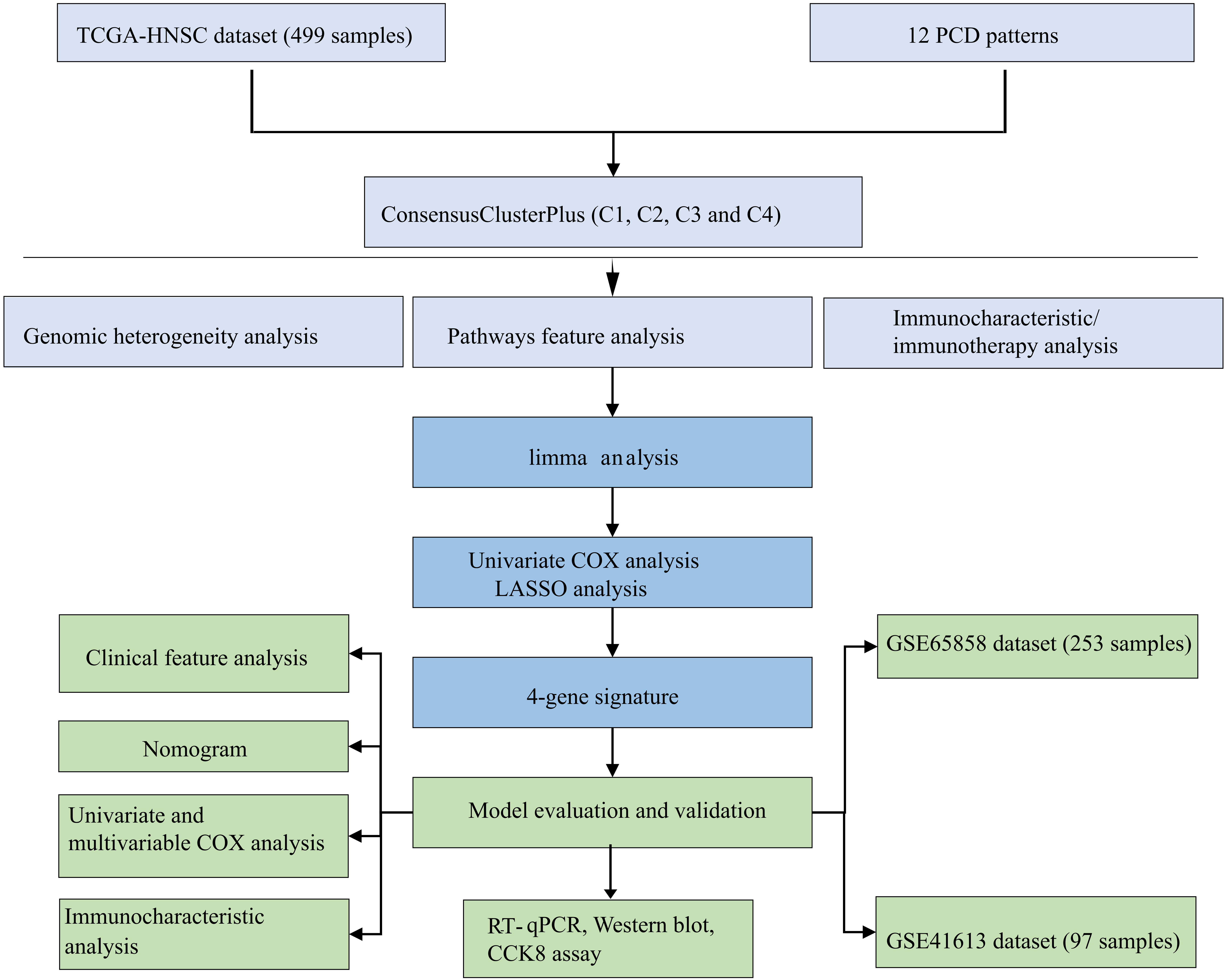
TCGA-HNSC dataset (499 samples)
12 PCD patterns
ConsensusClusterPlus (C1, C2, C3 and C4)
Genomic heterogeneity analysis
Pathways feature analysis
Immunocharacteristic/ immunotherapy analysis
limma analysis
Univariate COX analysis
LASSO analysis
Clinical feature analysis
4-gene signature
GSE65858 dataset (253 samples)
Nomogram
Univariate and multivariable COX analysis
Model evaluation and validation
Immunocharacteristic analysis
RT- qPCR, Western blot, CCK8 assay
GSE41613 dataset (97 samples)
