# Supplementary figures and images for: Head and neck squamous cell carcinoma-specific prognostic signature and drug sensitive subtypes based on programmed cell death-related genes
Source: PeerJ. 2023 Nov 21;11:e16364. doi: 10.7717/peerj.16364 (PMC10668860; doi:10.7717/peerj.16364)

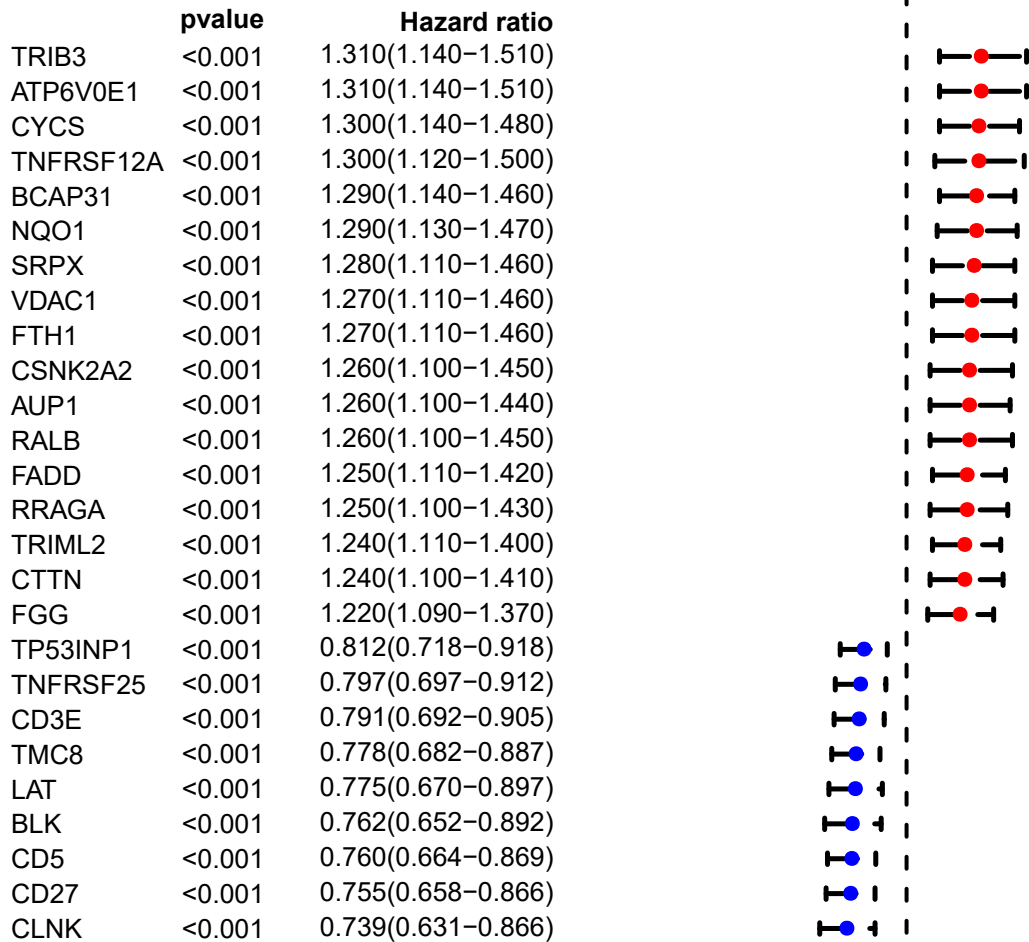

0.0 0.4 0.8 1.2

Hazard ratio

Supplement: Supplemental Information 2 [file peerj-11-16364-s002.pdf]

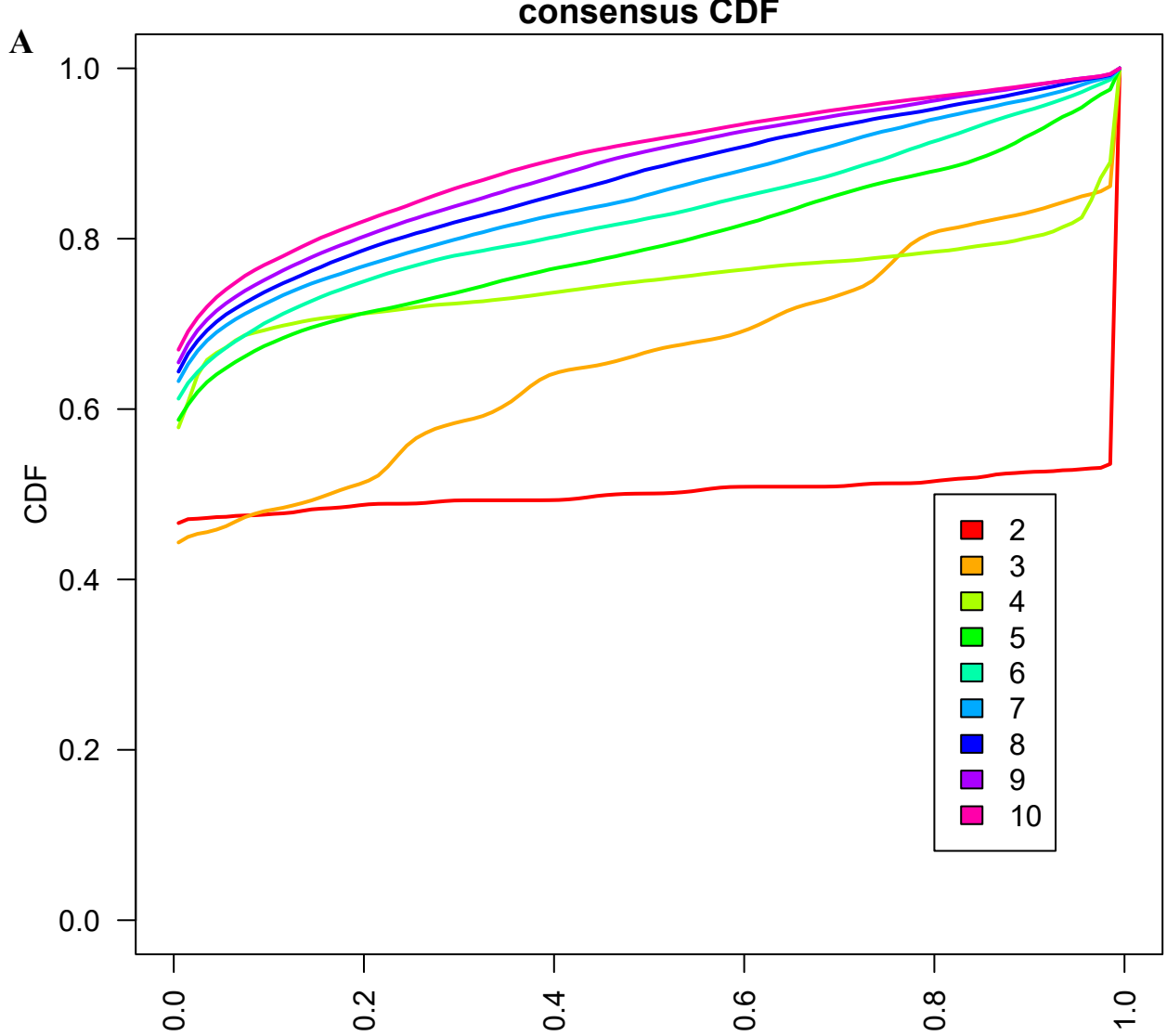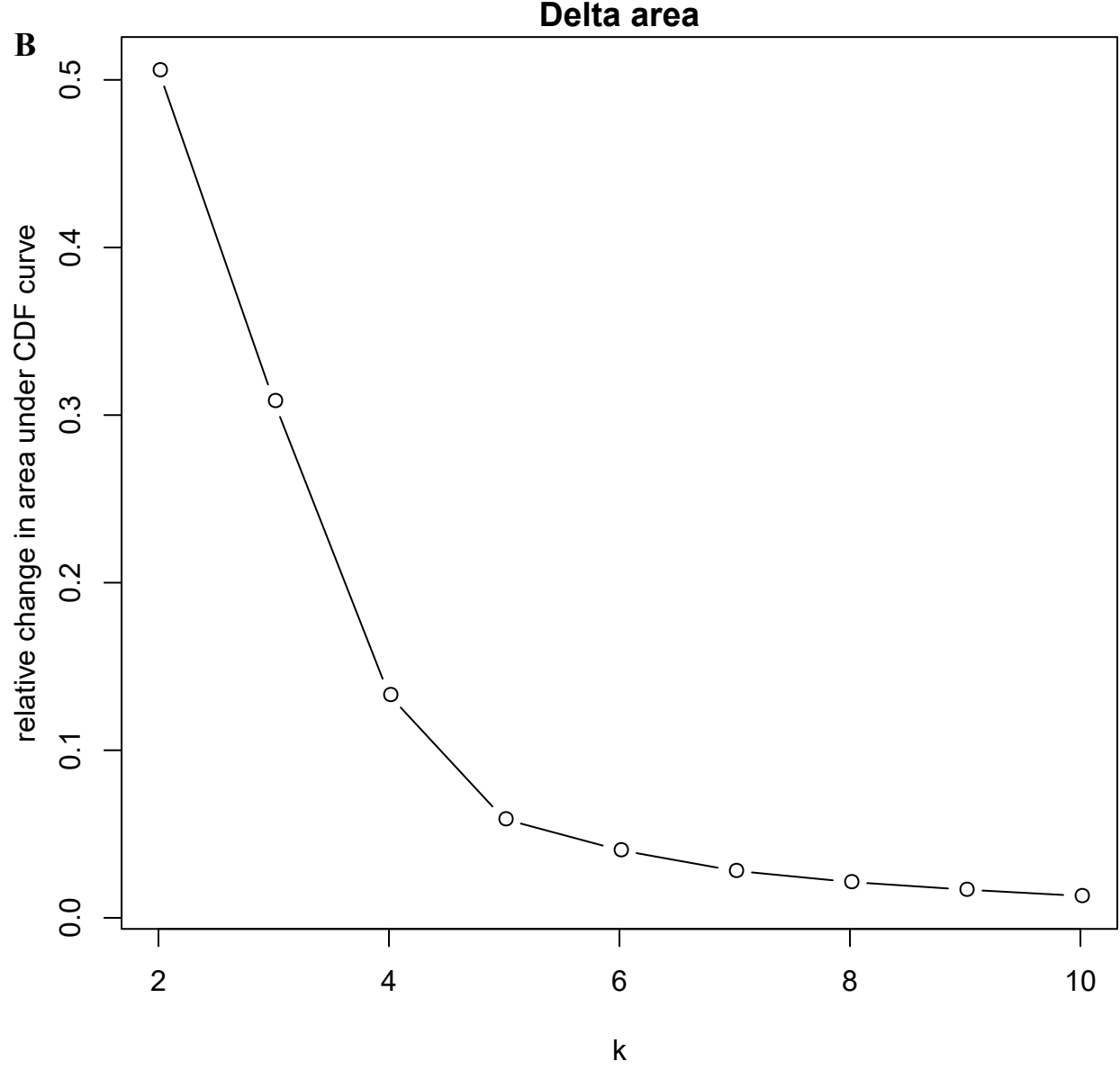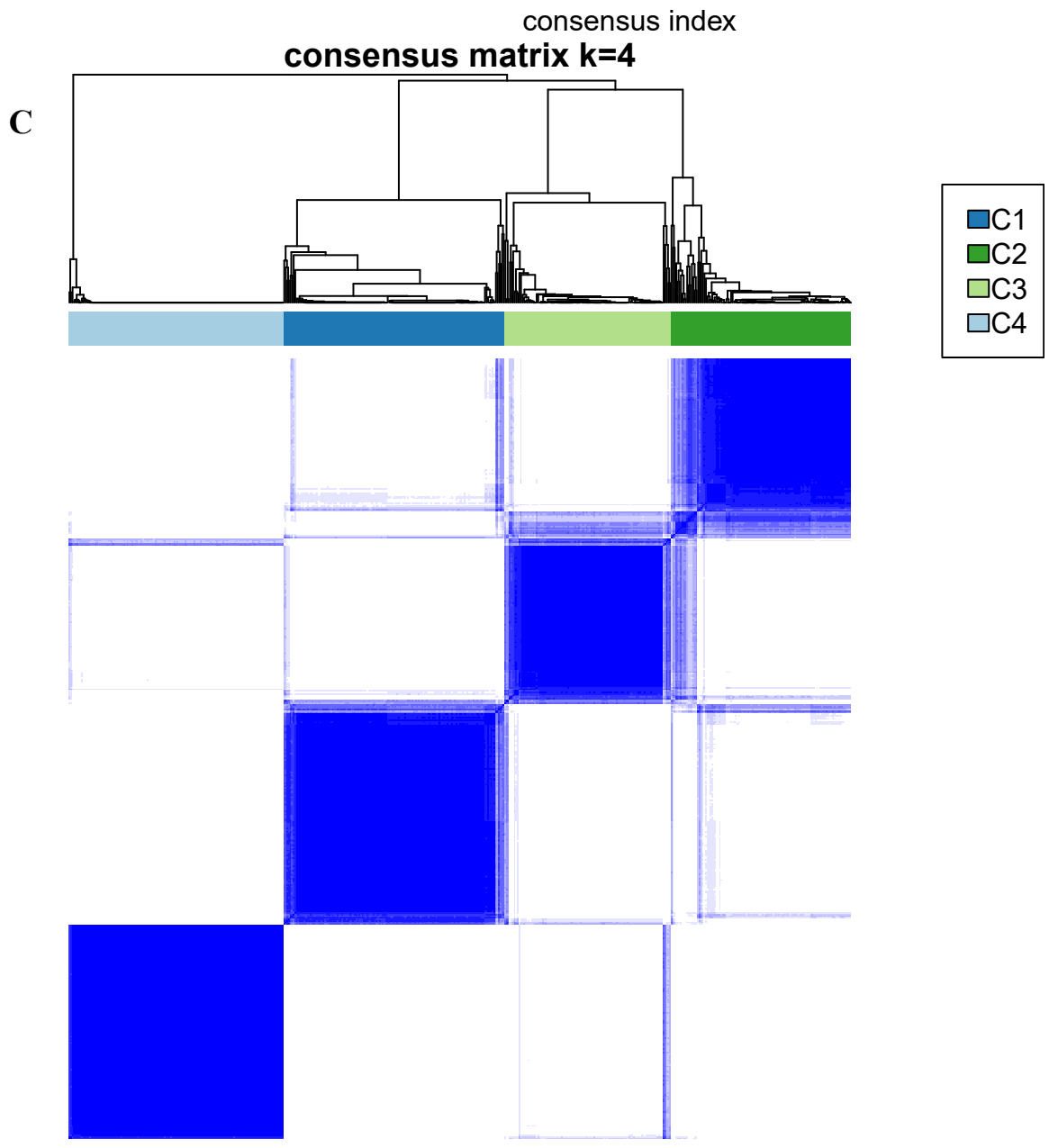

Supplement: Supplemental Information 3 [file peerj-11-16364-s003.pdf]

A

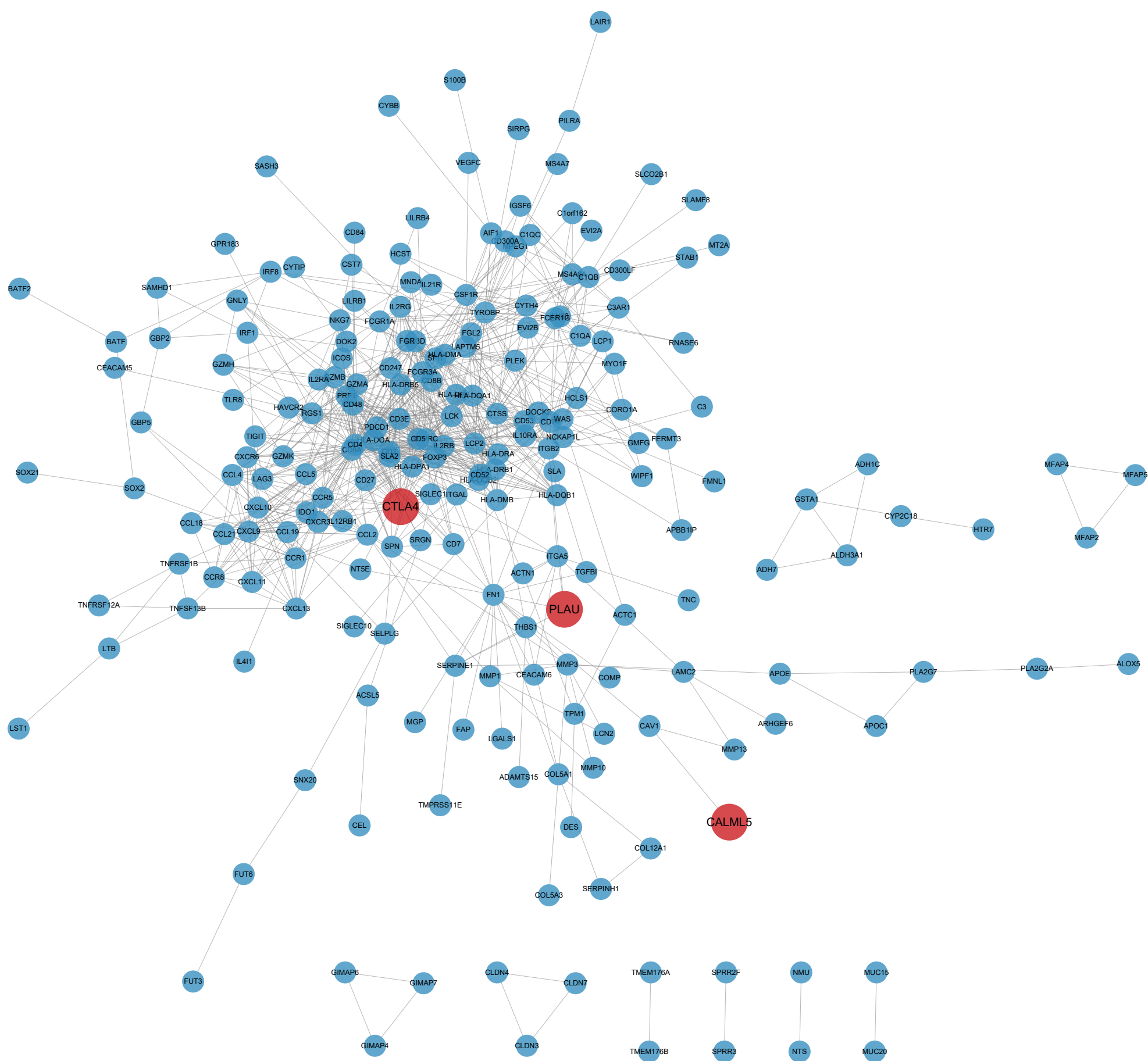

B

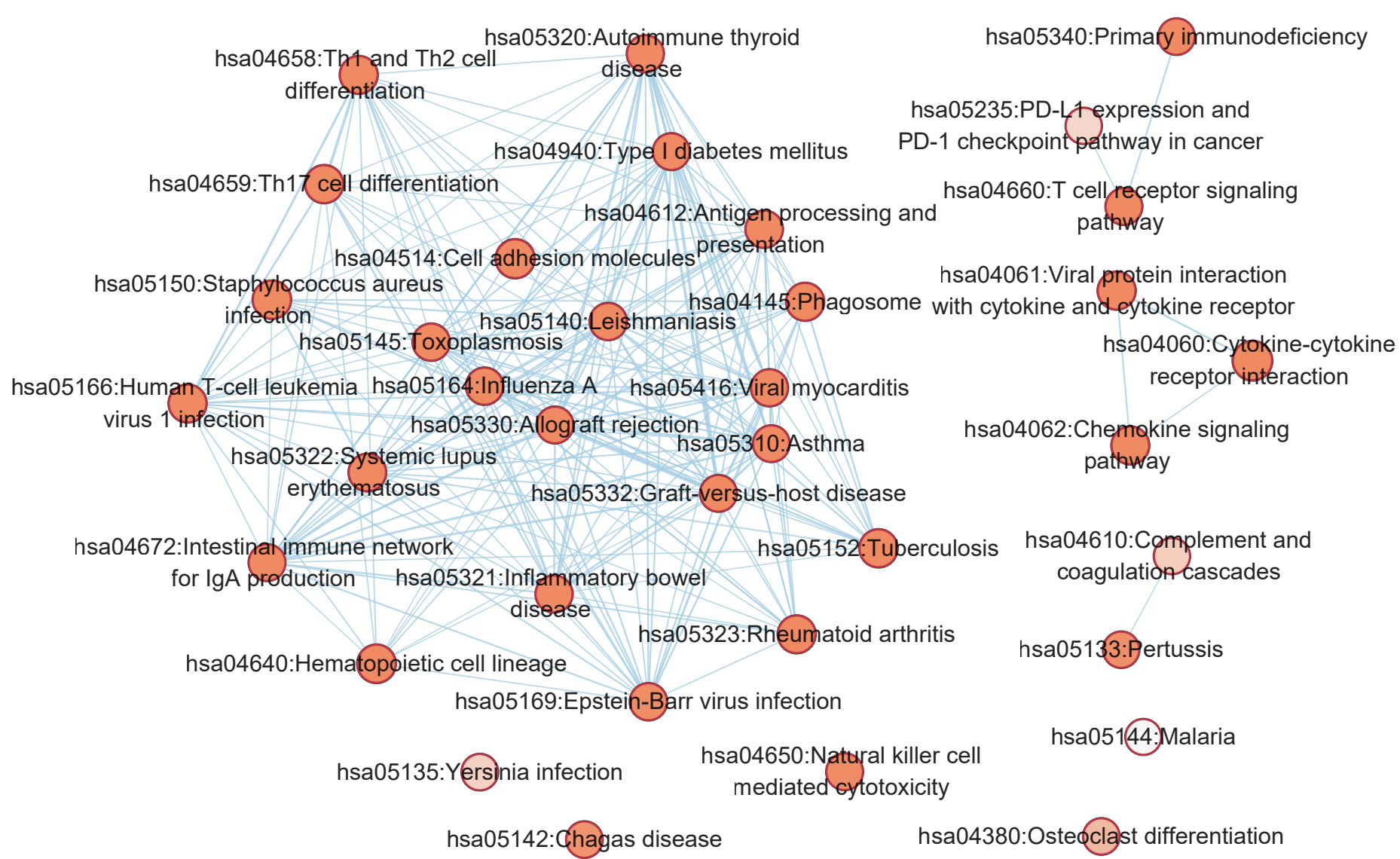

Supplement: Supplemental Information 4 [file peerj-11-16364-s004.pdf]
